# Role of angiogenesis-related lncRNAs in tumor microenvironment and prognosis of lung adenocarcinoma

**DOI:** 10.1016/j.gendis.2025.101700

**Published:** 2025-06-11

**Authors:** Lifeng Li, Yaqi Yang, Mengle Peng, Zhirui Fan, Xiaoran Duan, Ruyue Xue, Xuefeng Lv, Ming Cheng, Jie Zhao

**Affiliations:** a National Engineering Laboratory for Internet Medical Systems and Applications, The First Affiliated Hospital of Zhengzhou University, Zhengzhou University, Zhengzhou, Henan 450052, China; b Cancer Center, The First Affiliated Hospital of Zhengzhou University, Zhengzhou University, Zhengzhou, Henan 450052, China; c Medical School, Huanghe Science and Technology University, Zhengzhou, Henan 450000, China; d Fuwai Central China Cardiovascular Hospital, Internet Medical and System Applications of National Engineering Laboratory, Zhengzhou, Henan 450052, China; e Department of Clinical Laboratory, Henan No.3 Provincial People's Hospital, Zhengzhou, Henan 450006, China; f Department of Integrated Traditional and Western Medicine, The First Affiliated Hospital of Zhengzhou University, Zhengzhou University, Zhengzhou, Henan 450052, China; g Department of Clinical Laboratory, The Third Affiliated Hospital of Zhengzhou University, Zhengzhou, Henan 450052, China; h Department of Medical Information, The First Affiliated Hospital of Zhengzhou University, Zhengzhou University, Zhengzhou, Henan 450052, China; i Department of Pharmacy, The First Affiliated Hospital of Zhengzhou University, Zhengzhou University, Zhengzhou, Henan 450052, China

Long noncoding RNAs (lncRNAs), especially angiogenesis-related lncRNAs (ARLncs), are vital cancer biomarkers.^1^^,^^2^ This study explores their role in lung adenocarcinoma (LUAD), focusing on their influence on the tumor environment and angiogenesis. We identified 12 ARLncs to develop a prognostic signature independent of conventional indicators for LUAD patients. Notably, the low-risk group showed better outcomes, higher cytotoxic T-lymphocyte-associated protein 4 (CTLA4) expression, and improved response to CTLA4 checkpoint inhibitors. LINC00892 stood out as a key regulator of CTLA4 expression, linked to increased levels via vascular endothelial growth factor A (VEGFA). LINC00892 overexpression in LUAD cells boosted human umbilical vein endothelial cell (HUVEC) proliferation and migration by sponging miR-130b-3p and controlling VEGFA. This study introduces an innovative ARLncs-based prognostic model for LUAD, highlighting LINC00892's role in modulating CTLA4 expression through VEGFA, potentially guiding immunotherapy strategies for LUAD.

We screened 135 angiogenesis-related genes from the Gene Set Enrichment Analysis (GSEA) database and identified 2294 ARLncs through Pearson correlation analysis. Among these, 34 prognostic ARLncs were selected for further analysis (Fig. S1A). Box plot and heat map demonstrated that 18 ARLncs were up-regulated and 16 were down-regulated in tumor tissue compared with normal samples (Fig. S1B, S2A). Using consensus clustering, LUAD patients were grouped into two clusters based on ARLnc expression patterns, revealing distinct differences in M stage, T stage, clinical stage, and gender (Fig. S1C, S2B–D). Notably, ARLncs AL365181.2, AL365181.3, and AL59066.2 were particularly distinguishing, with higher expression in Cluster 1 (Fig. S1D). Cluster 1 patients exhibited poorer survival outcomes than Cluster 2 (Fig. S1E). Functional analyses highlighted Cluster 1's association with metabolic processes based on Gene Ontology and Kyoto Encyclopedia of Genes and Genomes pathway analyses (Fig. S2D, E). Investigation of immune checkpoint genes showed decreased levels of CTLA4 and T cell immunoglobulin and ITIM domain (TIGIT), and increased levels of programmed death ligand 1 (PD-L1) and hepatitis A virus cellular receptor 2 (HAVCR2) in tumor tissues compared with normal tissues (Fig. S1F, S2F, H, J). Expression of these genes varied significantly between Cluster 1 and Cluster 2, with higher expression of PD-L1, CTLA4, HAVCR2, and TIGIT in Cluster 2 (Fig. S1G, S2G, I, K). Correlation analysis between the 34 ARLncs and immune checkpoint genes identified notable associations, such as LINC00892 with CTLA4 and AC099850.4 with PD-L1 (Fig. S1H, S2L).

We used LASSO regression to identify 12 key ARLncs for a prognostic model (Fig. 1A, B). The risk score was computed using specific coefficients for each lncRNA. The receiver operating characteristic curve analysis indicated the values for the area under the curve of 0.790, 0.752, and 0.727 for predicting one-, two-, and three-year overall survival, respectively (Fig. 1C). This model showed better predictive accuracy than traditional factors like age, gender, and clinical stage (Fig. 1D). Validation split cohorts into high- and low-risk groups, revealing distinct expression patterns of the 12 ARLncs (Fig. 1E). Kaplan–Meier analysis confirmed shorter progression-free survival in high-risk patients (Fig. S3A), with higher risk scores correlating with increased mortality (Fig. S3B). Univariate and multivariate Cox regression analyses demonstrated independent prognostic value (Fig. S3D, E), while multivariate analysis highlighted T/N status and clinical stage for overall survival prediction. Evaluation using C-indexes and a nomogram (Fig. 1F, G; Fig. S3C) underscored the model's accuracy in predicting survival across patient subgroups (Fig. S4).

**Figure 1:**
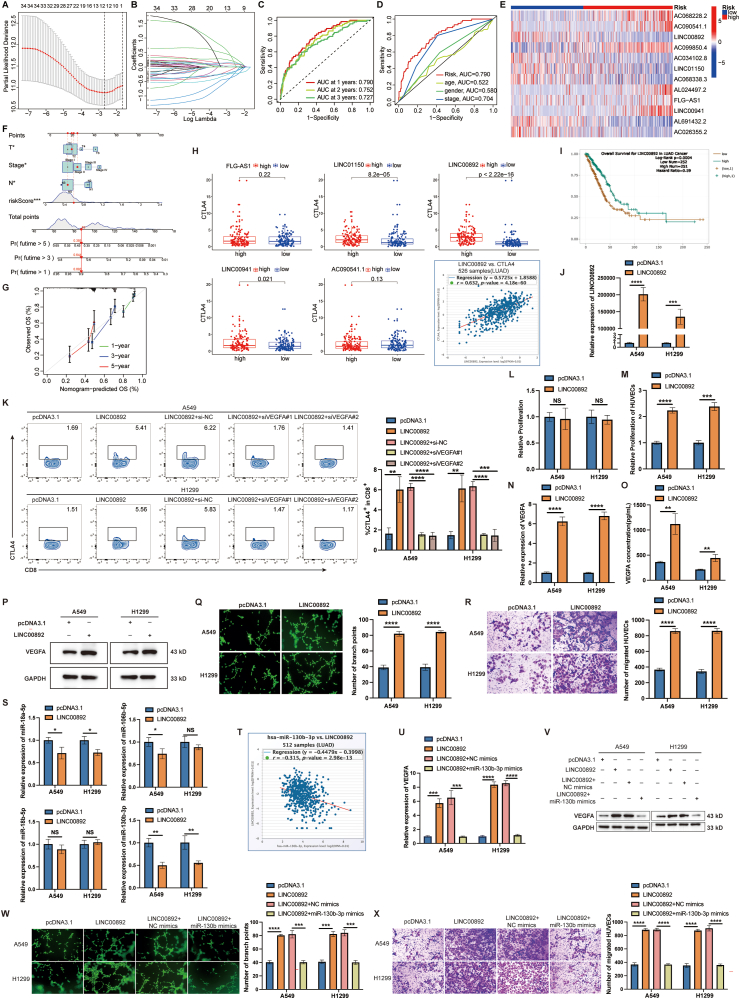
The construction of ARLncs prognostic model and the regulation of LINC00892 in lung adenocarcinoma (LUAD). **(A)** Selection of the optimal penalty parameter for LASSO regression. **(B)** LASSO regression analysis results. **(C)** The receiver operating characteristic (ROC) analysis depicting 1-year, 3-year, and 5-year survival risk profiles. **(D)** The ROC analysis evaluating risk, age, gender, and stage. **(E)** The heatmap illustrating the expression of model-associated ARLncs in high-risk and low-risk groups. **(F)** The nomogram integrating risk score, age, and tumor stage to predict the probability of 1-, 2-, and 3-year overall survival (OS). **(G)** Calibration curves for 1-, 3-, and 5-year OS predicted by the nomogram. **(H)** Correlation analysis between cytotoxic T-lymphocyte associated protein 4 (CTLA4) and FLG-AS1, LINC01150, LINC00941, AC090541.1, or LINC00892 in the TCGA cohort, and the correlation between the expression of LINC00892 and CTLA4 in LUAD tumor samples in the StarBase database (*n* = 526). **(I)** The correlation between the expression of LINC00892 and the prognosis of LUAD patients in the StarBase database (*n* = 503). **(J)** Quantitative reverse transcription PCR (RT-qPCR) analysis of LINC00892 expression levels after transfection with plasmids in A549 and H1299 cells. **(K)** Flow cytometry analysis was conducted on post-co-incubated human peripheral blood mononuclear cells (PBMCs) to detect CTLA4 expression in CD8^+^ T cells across different treatment groups. **(L)** CCK-8 assay detected the proliferation of A549 and H1299 cells after transfection with LINC00892 overexpression plasmids or pcDNA3.1 vector. **(M)** CCK-8 assay detected the proliferation of human umbilical vein endothelial cells (HUVECs) after culturing with supernatant from A549 or H1299 cells expressing LINC00892 or pcDNA3.1 vector. **(N, P)** RT-qPCR (N) and western blotting (P) assays detected the expression levels of VEGFA after transfection with LINC00892 overexpression plasmids or pcDNA3.1 vector in A549 and H1299 cells. **(O)** ELISA detected the levels of VEGFA in supernatant from A549 and H1299 cells after transfection with LINC00892 overexpression plasmids or pcDNA3.1 vector. **(Q)** Tube formation assay showed the effect of LINC00892 expression in LUAD cells on HUVECs. Scale bar, 50 μm. **(R)** Transwell assay showed the migration of HUVECs. Scale bar, 50 μm. **(S)** RT-qPCR analysis of the expression levels of miR-18a-5p, miR-106b-5p, miR-18b-5p, and miR-130b-3p following transfection with LINC00892 overexpression plasmids or pcDNA3.1 vector in A549 and H1299 cells. **(T)** Correlation analysis between miR-130b-3p and LINC00892 expression in LUAD patients from the StarBase database (*n* = 512). **(U, V)** RT-qPCR and western blotting analysis of VEGFA expression after co-transfection with LINC00892 overexpression plasmids and miR-130b-3p mimics in A549 and H1299 cells. **(W, X)** Tube formation assay (W) and transwell assay (X) assessed the impact of co-transfection with LINC00892 overexpression plasmids and miR-130b-3p mimics in LUAD cells on HUVECs. Scale bar, 50 μm. All experiments were conducted in triplicate, and data were presented as mean ± standard deviation. ∗*p* < 0.05, ∗∗*p* < 0.01, ∗∗∗*p* < 0.001, and ∗∗∗∗*p* < 0.0001; NS, not significant.

Our analysis of the tumor immune microenvironment revealed that the low-risk group had higher ESTIMATEScore (Fig. S5A), immuneScore (Fig. S5B), and stromalScore (Fig. S5C) compared with the high-risk group. CTLA4 expression was also higher in the low-risk group, whereas PD-L1 expression did not differ significantly (Fig. S5D, E). The low-risk group showed better response to CTLA4 inhibition, whereas PD-1 treatment did not confer significant benefit (Fig. S5F–I). In contrast, the high-risk group was associated with higher levels of macrophage M0 and CD4 memory-activated T cells, and lower levels of resting dendritic cells and CD4 memory-resting T cells (Fig. S5J, K), indicating distinct tumor microenvironment profiles between the groups. Additionally, we conducted sensitivity screening for chemotherapy drugs and identified over sixty clinically significant medications, including docetaxel, vinorelbine, doxorubicin, sunitinib, sorafenib, and paclitaxel (Fig. S6).

We analyzed tumor mutation burden in the high-risk and low-risk groups, identifying tumor protein P53 (TP53), titin (TTN), and mucin 16 (MUC16) as the top mutated genes and missense variations constituting the predominant mutation type (Fig. S7A, B). Lower tumor mutation burden correlated with poorer overall survival across different risk subgroups (Fig. S7C, D). The low-risk group showed significantly reduced tumor mutation burden compared with the high-risk group (Fig. S7E). Using the StarBase database, we mapped an lncRNAs-miRNAs-mRNAs network via a Sankey diagram, highlighting interactions among 8 lncRNAs, 14 miRNAs, and 5 mRNAs (Fig. S7F).

We validated expression of 12 ARLncs in BEAS-2B, A549, PC9, and H1299 cells. AC090541.1 and LINC00941 were up-regulated, while LINC00892, LINC01150, and FLG-AS1 were down-regulated in all LUAD cell lines (Fig. S8A), confirming our earlier findings. Our risk model accurately predicted scores for these cell lines (Fig. S8B), validated by transwell, wound-healing, and colony formation assays (Fig. S8C–H). BEAS-2B had the lowest risk score, while A549, PC9, and H1299 showed higher scores correlating with increased tumor cell proliferation and migration capacity. Our research shows that higher risk scores correlate with increased tumor cell proliferation and migration. Our prognostic model with the 12 ARLncs independently predicts LUAD prognosis.

In our study, we found that CTLA4 expression was higher in the low-risk group compared with the high-risk group, suggesting increased sensitivity to CTLA4 checkpoint inhibitors. Targeting CTLA4 has significantly enhanced the outcomes of certain cancer patients.^3^ However, there have been no reports on the relationship between lncRNAs and CTLA4. We investigated the correlation between CTLA4 and 5 selected ARLncs based on quantitative reverse transcription PCR results, identifying LINC00892 as having the strongest correlation, which was confirmed by StarBase (Fig. 1H). Elevated LINC00892 expression was associated with improved overall survival (Fig. 1I). To study LINC00892, we overexpressed it in A549 and H1299 cells using expression plasmids, confirming increased expression by quantitative reverse transcription PCR (Fig. 1J). Co-culture with peripheral blood mononuclear cells showed that LINC00892 overexpression notably increased CTLA4 levels (Fig. 1K). Investigating its role in angiogenesis, we silenced VEGFA in A549 and H1299 cells transfected with LINC00892, which reversed the CTLA4 elevation (Fig. 1K), suggesting that LINC00892 boosts CTLA4 via a VEGFA-dependent pathway.

We studied LINC00892 in LUAD and found that while it did not affect A549 and H1299 cell proliferation (Fig. 1L), it significantly enhanced HUVEC proliferation when their medium was conditioned by LINC00892-overexpressing cells (Fig. 1M). LINC00892 overexpression led to increased VEGFA expression and secretion (Fig. 1N–P), promoted tube formation (Fig. 1Q), and enhanced HUVEC migration (Fig. 1R). These results suggest that LINC00892 promotes VEGFA expression and HUVEC proliferation.

LINC00892 acts as a competing endogenous RNA to sequester miR-18b-5p, miR-130b-3p, miR-18a-5p, and miR-106b-5p (Fig. S7F). Among these, it has the strongest regulatory effect on miR-130b-3p, as confirmed by quantitative reverse transcription PCR (Fig. 1S) and StarBase analysis (Fig. 1T). By sponging miR-130b-3p, LINC00892 up-regulated VEGFA expression at both the mRNA and protein levels in A549 and H1299 cells (Fig. 1U, V). Co-culture experiments with HUVECs further showed that LINC00892-induced increases in tube formation and HUVEC migration were reversed by miR-130b-3p mimics treatment (Fig. 1W, X). This suggests that LINC00892 promotes angiogenesis by regulating VEGFA expression through miR-130b-3p *in vitro*.

In summary, we have developed a new prognostic model using 12 ARLncs that accurately predicts outcomes in LUAD patients. Our findings show that LINC00892 boosts CTLA4 expression via VEGFA and regulates VEGFA through miR-130b-3p, shedding light on lncRNAs' role in LUAD and suggesting potential for targeted therapies.

## CRediT authorship contribution statement

**Lifeng Li:** Data curation, Funding acquisition, Methodology, Writing – original draft, Writing – review & editing. **Yaqi Yang:** Data curation, Methodology, Writing – original draft, Writing – review & editing. **Mengle Peng:** Data curation, Supervision, Writing – review & editing. **Zhirui Fan:** Formal analysis. **Xiaoran Duan:** Data curation. **Ruyue Xue:** Methodology. **Xuefeng Lv:** Data curation. **Ming Cheng:** Data curation. **Jie Zhao:** Conceptualization, Funding acquisition, Supervision.

## Funding

This work was supported by the National Science Foundation of China (No. 32370976), Henan Key Laboratory of Chronic Disease Management (China) (No. HMKF202103), State Key Laboratory of Pathogenesis, Prevention and Treatment of High Incidence Diseases in Central Asia Fund (China) (No. SKL-HIDCA-2022- JZ5), Funding for Scientific Research and Innovation Team of The First Affiliated Hospital of Zhengzhou University, Henan, China (No. ZYCXTD2023005), and Wu Jieping Medical Foundation Special Fund for Targeted Cancer Research (China) (No. 320.6750.2023-02-1).

## Conflict of interests

The authors declared no competing interests regarding this study's publication.

## Figures and Tables

**figs1:**
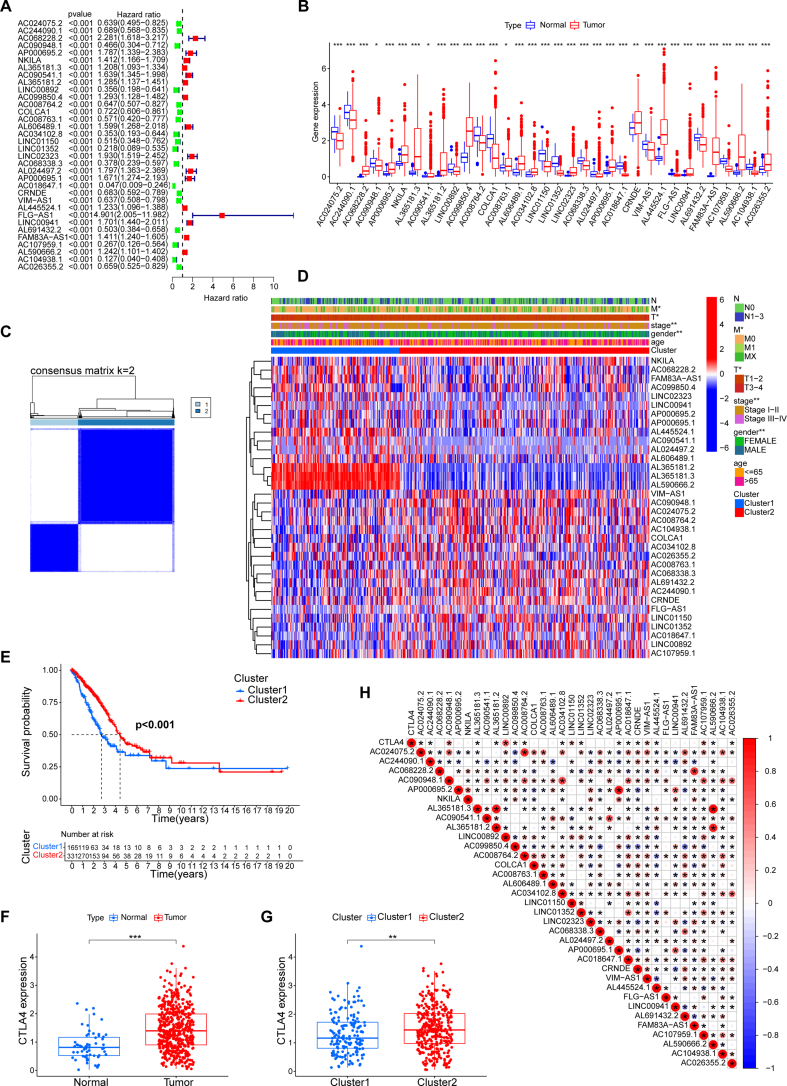


**figs2:**
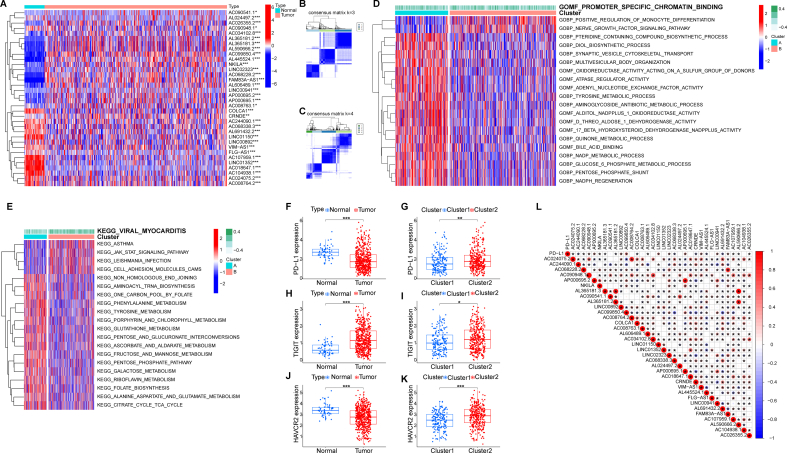


**figs3:**
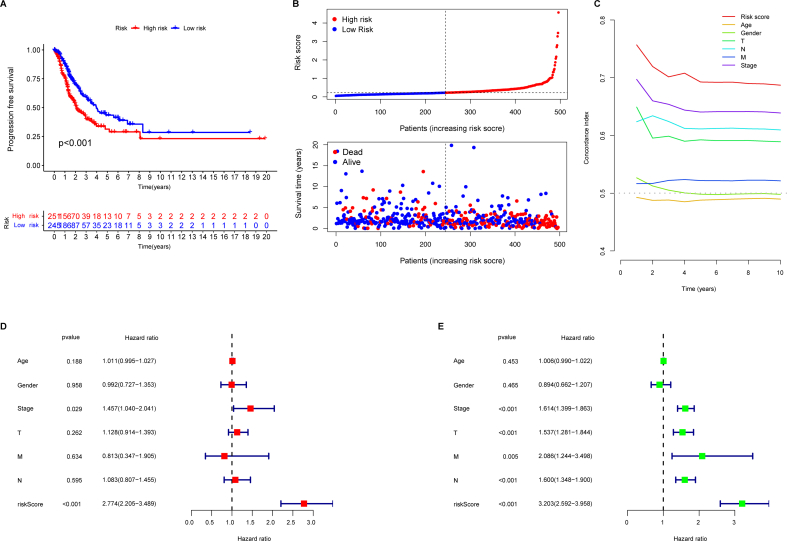


**Figure:**
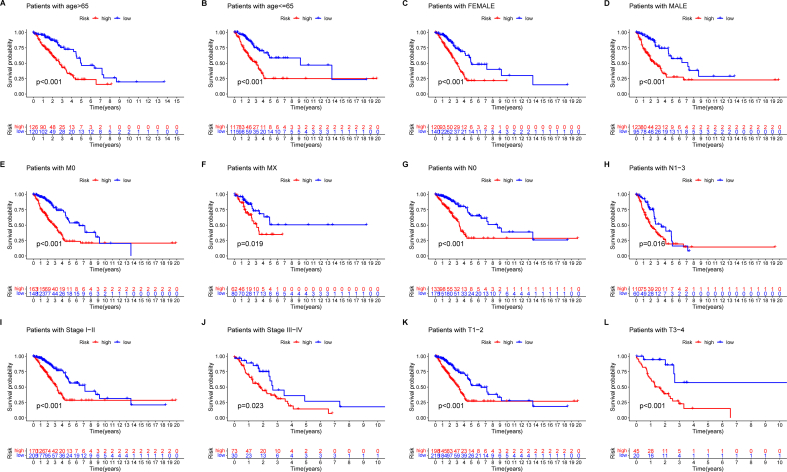
Figure S4 Validation of the model in different clinical sets. **(A**–**L)** Kaplan–Meier survival curve analysis demonstrated the model's predictive capability in various clinical groups, categorized by age (A, B), gender (C, D), M stage (E, F), N stage (G, H), clinical stage (I, J), and T stage (K, L).

**Figure:**
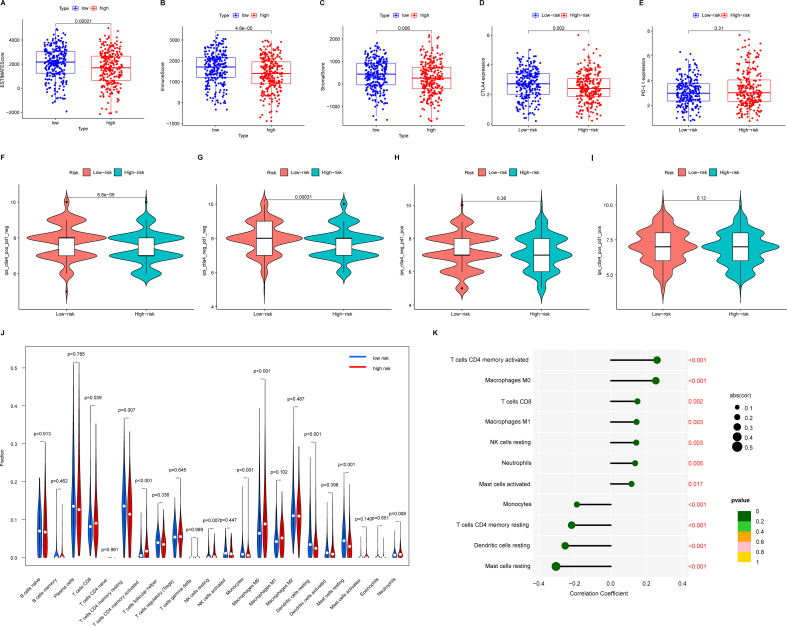
Figure S5 Analysis of tumor immune microenvironment (TIME) between two groups. **(A**–**E)** Differential analysis of ESTIMATE score (A), immune score (B), stromal score (C), cytotoxic T-lymphocyte associated protein 4 (CTLA4) expression (D), and programmed death ligand 1 (PD-L1) expression (E) between the two groups. **(F–I)** Sensitivity analysis of immunotherapy. **(J, K)** Differential analysis of immune cell infiltration.

**Figure:**
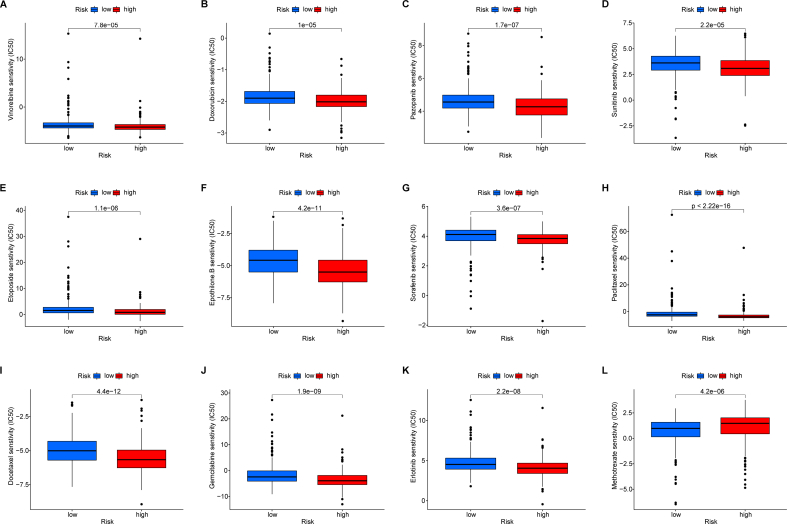
Figure S6 IC50 values of 12 commonly used chemotherapy drugs showed significant differences between high- and low-risk groups.

**Figure:**
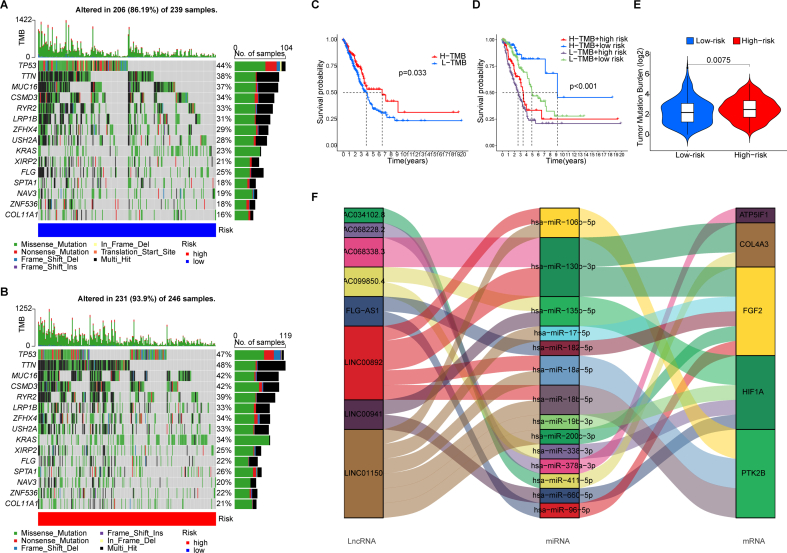
Figure S7 Tumor mutation burden (TMB) analysis between high- and low-risk groups and construction of ceRNA network. **(A, B)** The waterfall plot depicting the different TMB between high- and low-risk groups. **(C)** The survival analysis comparing high TMB with low TMB. **(D)** The survival analysis comparing the high-risk group with the low-risk group within the high and low TMB subgroups. **(E)** Analysis of TMB between high- and low-risk groups. **(F)** Construction of the ceRNA network.

**Figure:**
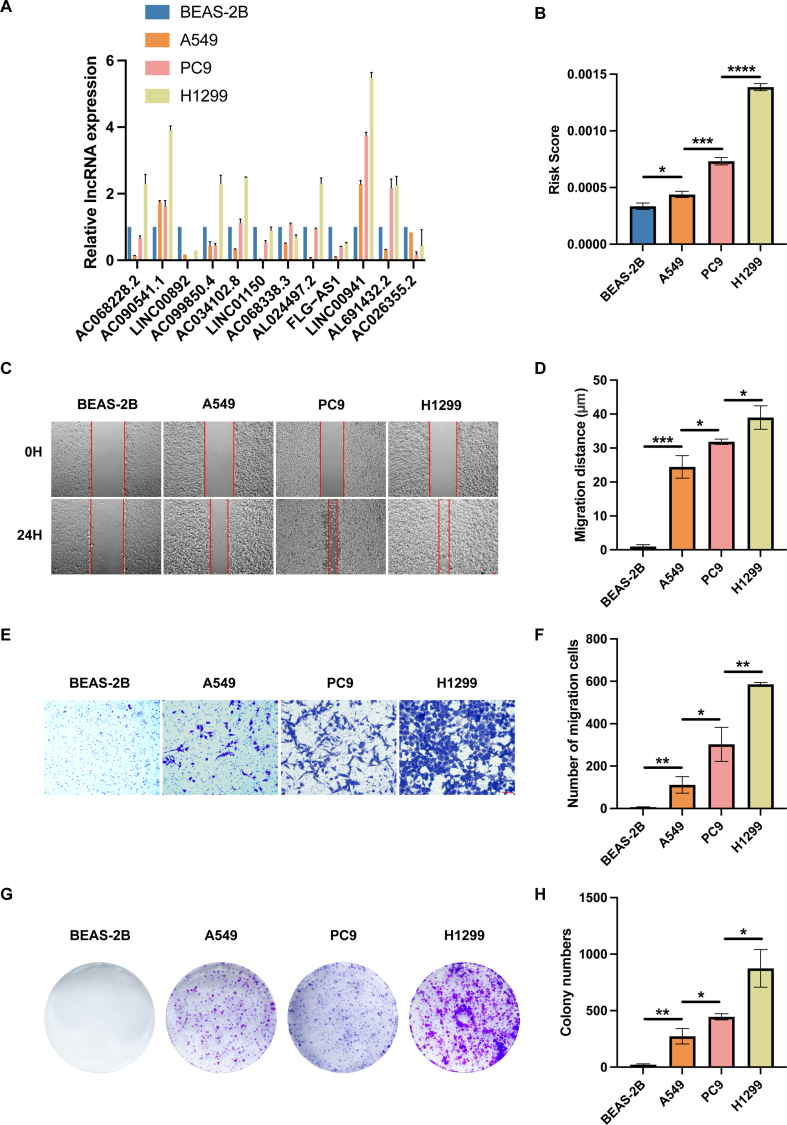
Figure S8 Validation of the risk signature via quantitative reverse transcription PCR and phenotypic experiments. **(A)** Quantitative reverse transcription PCR validation of the expression of 12 ARLncs in normal lung epithelium cell lines and human lung adenocarcinoma (LUAD) cell lines. **(B)** Assessment of the risk score in normal lung epithelium cell lines and human LUAD cell lines. **(C–H**) Results from (C, D) wound-healing assays, (E, F) transwell assays, and (G, H) colony formation assays in normal lung epithelium cell lines and human LUAD cell lines. Scale bar, 50 μm. All experiments were conducted in triplicate, and data were presented as mean ± standard deviation. ∗*P* < 0.05, ∗∗*P* < 0.01, ∗∗∗*P* < 0.001, and ∗∗∗∗*P* < 0.0001.
