# Supplementary material for: Role of angiogenesis-related lncRNAs in tumor microenvironment and prognosis of lung adenocarcinoma
Source: Genes Dis. 2025 Jun 11;12(6):101700. doi: 10.1016/j.gendis.2025.101700 (PMC12281221; doi:10.1016/j.gendis.2025.101700)
Supplement: Multimedia component 1 [file mmc1.docx]

# Material and Methods

## Data Acquisition and Processing

A 135-gene set related to angiogenesis was extracted from the Gene Set Enrichment Analysis (GSEA) database. The requisite clinical date was sourced from the Cancer Genome Atlas (TCGA) database (https://tcga-data.nci.nih.gov/tcga/). The patients were randomly allocated to either the training set or the testing set in a 1:1 ratio. Further details can be found in Supplementary Table 1.

## Screening of ARLncs

Employing pearson correlation analysis to assess the relationship between the angiogenesis-associated genes and lncRNAs. The following criteria were employed to identify ARLncRNAs: an absolute correlation coefficient exceeding 0.4 and a P-value of less than 0.001 (|R| > 0.4 and P < 0.001). The next step was to conduct univariate Cox regression analysis in order to identify prognostic ARLncs(P < 0.001). In total, 34 ARLncs were selected for additional examination.

## Clustering analysis

On the basis of the expression levels of ARLncs, a consensus clustering approach was conducted to stratify patients diagnosed with LUAD into distinct subgroups. Through the analysis, it was determined that k=2 provided the optimal clustering stability, as indicated by the similarity observed in the expression levels of ARLncs across a range of k values from 2 to 12. Consequently, the patients with LUAD were subsequently divided into cluster 1 and cluster 2.

## Functional Annotation of ARLncs

In order to identify the significantly enriched functions within each cluster, the Gene Ontology (GO) and Kyoto Encyclopedia of Genes and Genomes (KEGG) pathway analyses were conducted utilizing the limma package. Enrichment terms and pathways with a p-value less than 0.05 are supposed to have statistical significance in their enrichment.

## Structure and Evaluation of ARLncs Prognostic Model

LASSO regression model analysis was utilized to identify 12 ARLncs and then construct a novel prognostic signature. The formula for calculating the risk score was as follows: risk score = ∑(Coef(i) * x(i)), in which Coef(i) denotes the estimated regression coefficient, while x(i) represents the expression value associated with each ARLnc. The median risk score enabled LUAD patients to be categorised as high-risk or low-risk individuals. To ascertain the magnitude of the disparity in the groups' survival rates, receiver operating characteristic (ROC) curves were constructed and the area beneath the curve (AUC) was determined. Furthermore, the C-indexes were computed in order to assess the predictive performance of the model.

## Nomogram Generation

Nomograms for predicting 1-, 3- and 5- year overall survival (OS) were generated using R package RMS, considering independent prognostic factors including T-, N-, clinical stage and the predicted risk score. The nomogram provides a visual representation of the prognostic model, allowing for the estimation of individualized survival probabilities. A calibrating curve was generated to assess the reliability and accuracy of the nomogram.

## Tumor Immune Microenvironment (TIME) Analysis

We utilized the "estimate" package to determine the differences in immune infiltration, including the immuneScore, ESTIMATEScore, and stromalScore, between the high-risk group and the low-risk group. Additionally, we analyzed the infiltrated 22 types of immune cell and obtained results concerning the various infiltrated types of immune cell in the tumors by applying CIBERSORT. Furthermore, to examine the association of the risk score and the infiltration of immune cell, Pearson correlation analysis was performed. The clinical TCIA data of LUAD were downloaded from The Cancer Immunome Atlas (TCIA) database for the purpose of evaluating the potential therapeutic response of patients to CTLA4 blockade therapies.

## Tumor Mutation Burden (TMB) Analysis and competing endogenous RNAs (ceRNA) Network Mapping

The difference in TMB across groups was evaluated using the "ggpubr" package. For the investigation of the potential interactions of lncRNAs and miRNAs, we utilized the StarBase database. To assess the correlation of the levels of the expression of the lncRNAs and mRNAs, Pearson's correlation coefficient was employed. We considered correlation coefficients (|cor|) greater than 0.2 and p-values lower than 0.05 to be highly significant. Subsequently, we mapped a ceRNA network on the basis of these findings.

## Cell Cultivation and Transfection

The BEAS-2B cell line, originating from normal lung epithelium, as well as the human LUAD cell lines A549, PC9, and H1299, and Human Umbilical Vein Endothelial Cells (HUVEC), procured from the National Collection of Authenticated Cell Cultures and maintained by the Precision Laboratory of the National Telemedicine Center at The First Affiliated Hospital of Zhengzhou University, were cultured in Dulbecco's Modified Eagle Medium (DMEM) supplemented with 10% fetal bovine serum (FBS) (Thermo Fisher Scientific, USA) and 1% penicillin-streptomycin (Thermo Fisher Scientific, USA), at 37°C in a 5% CO2 atmosphere. GenePharma designed and synthesised plasmids overexpressing LINC00892 based on the pcDNA3.1 vector and miR-130b-3p mimics. Tsingke Biotech designed and manufactured siRNAs targeting VEGFA. LUAD cells were plated onto 6-well plates and subjected to transfection on the subsequent day upon reaching a cell density of 40%-50%. Lipofectamine™2000 reagent (Invitrogen, USA) was utilized for transfection following the manufacturer's guidelines. Cells were collected 48 hours post-transfection for subsequent experiments. See Supplementary Table 2 for sequences of siRNAs and mimics.

## Isolation of RNA and Real-time Quantitative Polymerase Chain Reaction (RT-qPCR)

TRIzol reagent (Invitrogen, Carlsbad, CA, USA) was used for extraction of total RNA from normal and LUAD cells. For lncRNA and mRNA, the synthesis of complementary DNA was performed with Evo M-MLV RT Premix for PCR, for miRNA, miRNA 1st Strand cDNA Synthesis Kit (Accurate Biology, China) was used for cDNA synthesis. RT-qPCR was performed using SYBR Green Premix Pro Taq HS qPCR kits (Rox plus) (Accurate Biology, China). The internal reference genes were beta-actin and U6. Supplementary Table 3 showed the primer sequences.

## Transwell, Wound-healing and Colony Formation Assays

To assess the migratory ability of the cells, transwell and wound healing assays were carried out. Cells were prepared in serum-free medium at a density of 2 × 10^5^ cells/ml (200 μL) for the transwell assay and were added to the upper chamber. 600 μL of DMEM with 10% FBS was added to the lower chamber. The cells were then hatched under standard culture conditions for 24 hours. Cells were seeded into six-well plates and scraped with a pipe tip when they reached approximately 100% confluence for the wound healing assay. Cells were washed three times with PBS and serum-free maintained for 24 hours. Cell migration images were taken microscopically at 0 and 24 hours. To evaluate cell proliferation, a colony formation assay was conducted. The cells were seeded (1000 cells per well) in six-well plates and grown for a period of 10 days. After washing twice with PBS, the plates were fixed in 4% paraformaldehyde for 30 minutes and stained with 0.1% crystal violet for 30 minutes. All experimental results were characterised by means of Image J software.

**Tube Formation Assay**

Pre-cooled extracellular matrix gel was dosed into the wells of a new 96-well plate and cultured at 37°C in a cell culture incubator for 30 minutes to form a gel. HUVECs (1 × 10^4^ cells/well) suspended in conditioned medium were plated into individual wells and cultured in 5% CO2 at 37°C for 6-8 hours. Next, The culture medium was exchanged with calcein-AM-containing growth medium and further incubated for 20 minutes to allow for calcein staining. After staining, the wells were gently washed with PBS by shaking. Images of tube formation were captured using an inverted microscope at a 100x magnification, and tube formation was analyzed and measured using image J.

**Enzyme Immunoassay (ELISA)**

An ELISA assay kit (Dakewe) was employed to measure the VEGFA concentration in the culture medium of LUAD cells, adhering to the manufacturer's guidelines. The absorbance at 450 nm was read by means of a microplate reader.

**Cell Counting Kit-8 (CCK-8)**

Transfected A549 and H1299 cells, as well as conditioned medium-treated HUVEC cells, were trypsinized, counted, and plated at 3×10^3^ cells per well in a 96-well plate. Cells were cultured in a cell culture incubator for 24 hours at 37°C, 5% CO2. After washing the wells with PBS, 100 μL of fresh culture medium containing 10 μL CCK-8 reagent was given to the individual wells. Plates were allowed to incubate for 1 hour in a cell culture incubator. Subsequently, each sample was then absorbance measured at 450nm by microtitre plate reader.

**Western blotting**

RIPA buffer (Beyotime) with inhibitors of protease and phosphatase was used to lyse LUAD cells. The BCA assay kit served to quantify the protein concentration. Protein samples were equally loaded onto 10% SDS-PAGE gels and then transferred onto polyvinylidene fluoride membranes. Following blocking with 5% skim milk in TBST at room temperature for 2 hours, the membranes underwent overnight incubation at 4°C with primary antibodies. Subsequently, horseradish peroxidase-conjugated secondary antibodies (Proteintech) were applied for 2 hours at room temperature. Protein expression was visualized using ECL chemiluminescence reagent. The primary antibodies utilized were GAPDH (1:3000, #60004-1-Ig, Proteintech) and VEGFA (1:1500, #66828-1-Ig, Proteintech).

**Flow Cytometry**

Harvested human PBMCs after co-culture were stained with FITC-conjugated human CD3 antibodies, PE-conjugated human CD8 antibodies, and APC-conjugated human CTLA4 antibodies, following manufacturer's instructions (BioLegend, USA). Subsequently, samples were collected using a flow cytometer (BD FACSCanto II, USA), and analysis of data was by means of FlowJo in accordance with the manufacturer's instructions.

## Statistical Analysis

R version 3.6.1 and associated packages were used for all statistical analyses. Pearson correlation analysis, LASSO regression model analysis, Kaplan-Meier analysis and COX analysis were applied to the selection of ARLncs and assessment of the efficiency of the signature. P<0.05 has been set as statistical significance for all data.
