# Supplementary material for: Role of angiogenesis-related lncRNAs in tumor microenvironment and prognosis of lung adenocarcinoma
Source: Genes Dis. 2025 Jun 11;12(6):101700. doi: 10.1016/j.gendis.2025.101700 (PMC12281221; doi:10.1016/j.gendis.2025.101700)
Supplement: Multimedia component 2 [file mmc2.docx]

**Table S1** Clinical characteristics of all LUAD patients.

| **Character** | **Training cohort** | **Testing cohort** | **Total cohort** |
| --- | --- | --- | --- |
| Age(year)  ≤65  >65  Unknown | 122(49.2%)  121(48.8%)  5(2.0%) | 114(46.0%)  129(52.0%)  5(2.0%) | 236(47.6%)  250(50.4%)  10(2.0%) |
| Gender  Female  Male | 130(52.4%)  118(47.5%) | 137(55.2%)  111(44.8%) | 267(53.8%)  229(46.2%) |
| Clinincal stage  I-II | 186(75%) | 198(79.8%) | 384(77.4%) |
| III-IV  Unknown | 58(23.4%)  4(1.6%) | 46(18.5%)  4(1.6%) | 104(21.0%)  8(1.6%) |
| T stage  T1-2  T3-4  Tx | 212(85.5%)  35(14.1%)  1(0.04%) | 217(87.5%)  29(11.7%)  2(0.8%) | 429(86.5%)  64(12.9%)  3(0.6%) |
| N stage  N0  N1-3  Nx  Unknown | 157(63.3%)  86(34.7%)  5(2.0%)  0 | 163(65.7%)  78(31.5%)  6(2.4%)  1(0.4%) | 320(64.5%)  164(33.1%)  11(2.2%)  1(0.2%) |
| M stage  M0  M1  Mx  Unknown | 174(70.2%)  16(6.4%)  57(23.0%)  1(0.4%) | 153(61.7%)  9(3.6%)  83(33.5%)  3(1.2%) | 327(65.9%)  25(5.0%)  140(28.2)  4(0.8%) |
